# Apexum: A Minimum Invasive Procedure

**DOI:** 10.5005/jp-journals-10005-1113

**Published:** 2011-04-15

**Authors:** Deepak Raisingani

**Affiliations:** Associate Professor, Department of Conservative Dentistry and Endodontics, Mahatma Gandhi Dental College and Hospital Jaipur, Rajasthan, India

**Keywords:** Apexum, Healing, Minimally invasive, Periapical lesions.

## Abstract

The new Apexum procedure (Apexum Ltd, Or-Yehuda, Israel) is based on a minimally invasive removal of periapical chronically inflamed tissues through a root canal access. Apexum procedure (a novel method that allows for the removal or debulking of periapical tissues without using scalpels, periosteal elevators, or sutures) results in significant less postoperative discomfort or pain than conventional root canal treatment or than reported for conventional apical surgery. The removal or debulking of the periapical inflamed tissues, using the Apexum procedure, seems to enhance healing kinetics with no adverse events.

## INTRODUCTION

Lesions of apical periodontitis represent an inflammatory response to bacterial infection of the root canal. Periapical radiolucencies is the most pronounced clinical hallmark of these lesions. Most, but not all, periapical lesions will heal in response to properly performed endodontic treatment. However, an evidence-based estimation to assess the healing potential cannot be performed before 12 months after surgery. In an extensive study, 0rstavik concluded that, at 6 months, only 50% of the cases that will eventually heal show clear signs of healing (advanced and complete healing), and at 12 months, 88% of the lesions that will eventually heal show clear signs of healing.^1^ This may imply that a case should ideally be followed for 12 months before the tooth may be considered a safe abutment.^2^

Such a time schedule is difficult to follow in everyday clinical practice because both the dentist and patient are eager to finish the case with a permanent restoration as soon as possible. The prolonged healing process of many periapical lesions has been attributed to the activated macrophages in the lesion that may maintain their state of activation long after the initial cause of their activation has been eliminated by root canal treatment; namely, the activation state may outlive its useful purpose and become a burden by inhibiting resolution of the lesion. The healing of similar lesions after apical surgery is much faster.^3^

Kvist and Reit^4^ have shown that surgically treated lesions of apical periodontitis healed during the first 12 months with significantly enhanced kinetics compared with those treated with nonsurgical retreatment. This was true even though both groups had similar healing rates over longer time periods. Fast healing of periapical lesions after apical surgery is a common clinical observation. These observations may indicate that surgical removal of the chronically inflamed periapical tissue may allow a fresh blood clot to form, which will then organize into an uncommitted granulation tissue and allows for faster healing. Performing apical surgery on every case with a periapical lesion will most likely enhance healing kinetics. Nevertheless, it can hardly be justified because surgery has repercussions for the well-being of the patient; swelling, pain and discomfort are among the expected side effects.^5^ Furthermore, many anatomic locations preclude apical surgery either because of inaccessibility or risk to adjacent structures. In accordance, the American Association of Endodontists recommends performing apical surgery only in cases that cannot be treated otherwise.^6^

Recently, a novel method was introduced that allows for the removal or debulking of periapical tissues without using scalpels, periosteal elevators or sutures.^7^ This method is based on a device that removes the chronically inflamed periapical tissues through a root canal access by a procedure that is minimally invasive compared with open-flap apical surgery. The new technology (Apexum Ablator; Apexum Ltd, Or- Yehuda, Israel) has the possibility of providing some of the benefits of apical surgery without the drawbacks of the conventional surgical procedure. This technological advancement may allow for the application of such a protocol in many cases in which healing time is a critical factor.

## APEXUM DEVICES

**Fig. 1:**
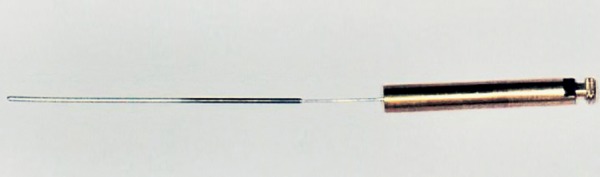
The Apexum NiTi Ablator in its sheath

**Fig. 2:**
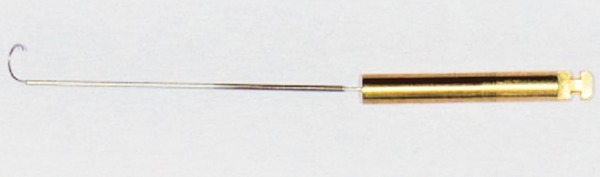
The Apexum NiTi Ablator pushed in and extruded from its sheath (arrow)

**Fig. 3:**
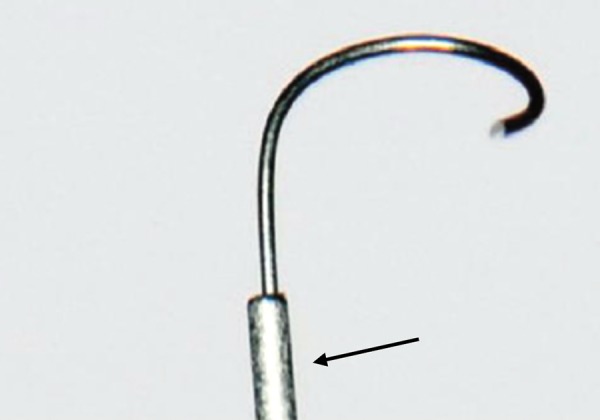
An enlarged view of the active part of the Apexum NiTi Ablator

The Apexum kit consists of two devices, the Apexum NiTi Ablator and Apexum PGA Ablator, designed to be used sequentially. Both instruments are for single use and were provided by Apexum Ltd. The Apexum NiTi Ablator consists of a specially preshaped Nitinol wire. One end is bent and is designed to enter the periapical tissues through the root canal and apical foramen, whereas the other end has a latch-type connector to allow its operation by a low-speed contra-angle handpiece. The bent part is initially concealed in a straight superelastic Nitinol tube that serves as a sheath allowing its introduction up to the apical foramen (Fig. 1). When pushed, the wire emerges from its sheath and through the apical foramen and resumes its preshaped form (Figs 2 and 3). The special retrograde design of the bent part allows it to rotate in the periapical soft tissues at 200 to 250 rpm and coarsely grind them while being deflected from the surrounding bone (see Fig. 3). The Nitinol sheath is used first to allow the introduction of the prebent Nitinol wire to the apical foramen and second to allow unobstructed rotation of the wire in the root canal without twisting of the wire.

**Fig. 4:**
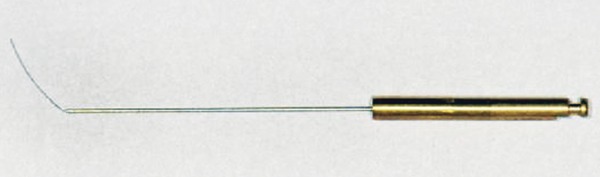
Apexum PGA Ablator

The second device is the Apexum PGA Ablator, built from a Nitinol shaft, equipped on one end with a latch-type connector to allow its operation by a low-speed contra-angle handpiece (Fig. 4). At the other end, a bioabsorbable filament is attached, which is designed to enter the peri-apical bony crypt and rotate at 5,000 to 7,000 rpm, turning the tissue that was initially minced with the NiTi Ablator into a thin suspension that may be flushed through the root canal.

### Apexum Procedure

**Fig. 5:**
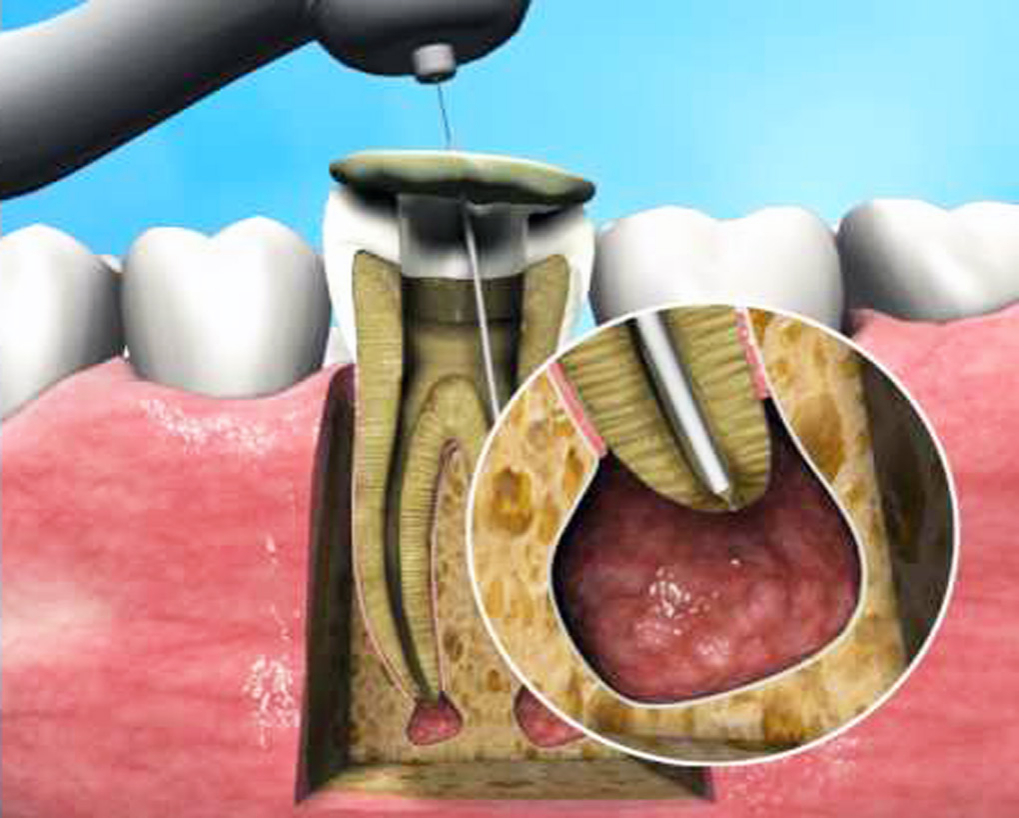
Apexum procedure

A #20 K-file is passed through the apical foramen and beyond the apex to verify patency. It was followed by a rotary #30 file that is passed 1 mm beyond the apical foramen, creating a passage. The Apexum NiTi Ablator is then inserted, while encased in its sheath, to the working length as established at the cleaning and shaping stage. The sheath is stabilized to the occlusal surface of the tooth using glass-ionomer cement (Fig. 5). The Nitinol filament is then pushed manually through the enlarged apical foramen and into the periapi-cal tissues. The filament is first rotated manually to verify mobility with no solid obstruction and then attached to a low-speed contra-angle handpiece.

**Fig. 6:**
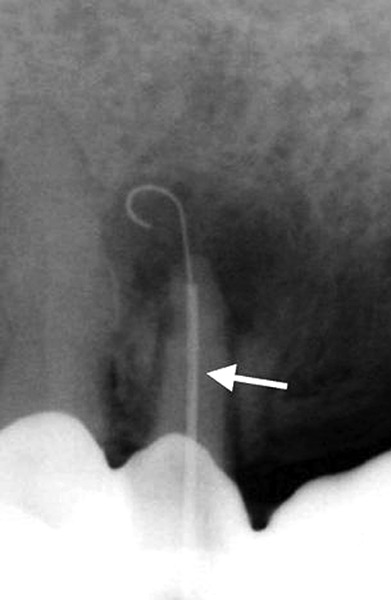
The Apexum NiTi Ablator in a periapical lesion. The NiTi Apexum Ablator fully extruded from its sheath (arrow) and into the periapical lesion

The NiTi Ablator is then rotated in the periapical tissues for 30 seconds at 200 to 250 rpm to initially mince the tissue (Fig. 6). The stabilizing glass-ionomer cement is then removed and the NiTi Ablator withdrawn from the root canal with its sheath to examine it for any mechanical damage or missing parts. The root canal is rinsed with sterile saline, and the Apexum PGA Ablator is manually inserted through the root canal and into the periapical tissues. It is then connected to a low-speed contra-angle handpiece and rotated for 30 seconds at 5,000 to 7,000 rpm to turn the minced tissues into a thin suspension. Next, it is withdrawn from the root and examined for any mechanical damage or missing parts. The tissue suspension is now washed out with sterile saline solution by using a syringe adapted with a 30-G blunt needle. The needle is passed through the enlarged apical foramen into the periapical space, and the solution is slowly and gently injected to flush the tissue suspension out. The cross-sectional area between the enlarged apical foramen and the outer surface of the needle is 3.4 times larger than that of the needle’s lumen. This facilitated an unobstructed backflow and prevented pressure build up in the periapical crypt. Nevertheless, special attention is given to visually monitor the backflow of the blood red suspension through the root canal continuously so that pressure build up did not occur in the periapical space. To allow for continuous monitoring, aspiration is performed at a distance from the access cavity so that the operator could visually evaluate the in- and outflow rates. The suspension will turn pale during the process, and the flushing is stopped and the needle removed when clear solution appeared. The root canal is then dried with sterile paper points and obturation conducted followed by a glass-ionomer cement temporary filling and a postoperative radiograph.

The Apexum procedure is performed under local anesthesia, provided in a manner similar to that used for tooth extraction or surgical intervention. With some experience, it will take an additional 7 to 10 minutes compared with a conventional root canal treatment.

## DISCUSSION

The new Apexum procedure represents a shift from the current endodontic paradigm. Foremost, it does not limit the endodontic intervention only to remove the cause (bacteria) and then allowing the host to heal at its own pace, and, furthermore, the device enters the periapical lesion far beyond the apical foramen, a process expected by many operators to result in a flare-up or severe symptoms.^8,9^ These results are rather surprising because the current paradigm is that the insertion of an endodontic instrument far beyond the apical foramen should be avoided by all means because it is likely to cause a ‘flare-up,’ a painful exacerbation of the periapical inflammatory process. It should be noted that the Apexum procedure is substantially different from simple overinstrumentation during root canal treatment. The last traumatizes the tissue and may also introduce bacterial antigens into a tissue containing immunoglobulins directed against these antigens and that is primed to respond to them (When this happens, acute inflammatory response with resulting edema is likely to occur in the periapical tissue, resulting in a flare-up.^8,9^

It is most likely that with the removal or major de-bulking of the periapical chronically inflamed tissues, the mechanisms that could otherwise lead to such a flare-up were also removed.

This may explain the quite uneventful postoperative clinical behavior with Apexum procedure. Whether the Apexum procedure is able to remove all of the periapical inflammatory tissue, as is usually attempted in conventional apical surgery, is doubtful. Most likely, the Apexum procedure removed all of this inflamed tissue in some lesions, whereas major debulking occurred in others. In any case, the procedure created conditions that allowed for faster healing of the lesions. Because the term ‘removal’ may be misinterpreted as ‘complete removal,’ the term ‘removal or debulking’ was chosen to describe the mechanical effect of the Apexum procedure on the peri-apical tissues.

*Other field of dentistry that can benefit from this technique is implantology:* When a broken-down tooth with a large periapical lesion has to be extracted and replaced by an implant, the implantologist is presented with a dilemma: if there was no bone defect around the apex, an immediate implant could be successfully placed. However, when there is a large periapical lesion and no bone to engage the implant’s apical part, augmentation will be required which becomes either a long and expensive story or a compromised procedure.

Of course, the implantologist would prefer to have bone at the depth of the extraction socket. It is precisely such bone augmentation that the Apexum procedure provides within a relatively short time. Furthermore, the implantologist will also be happy to preserve the alveolar socket walls for his implant. These requests can reproducibly be provided by the natural tooth which will be retained in its socket until the day of extraction.^10^

Thus, conducted in the same manner as in endodontic cases, the Apexum procedure will also serve as a preparatory stage for immediate implant placement in extraction sites of compromised teeth with large periapical lesions.

However, there are lot of controversies associated with this technique; widening of apical foramen to more than size 30, the extent of root canal preparation at its apical end is subject of much debate.

Other issues to be addressed are about management of procedural errors (instrument separation, etc.).

## CONCLUSION

So far the Apexum (minimally invasive procedure) resulted in significantly less postoperative discomfort or pain than conventional root canal treatment or than that reported for conventional apical surgery. The Apexum procedure has resulted in a significantly faster periapical healing as compared with conventional root canal treatment. The removal or debulking of the periapical inflamed tissues, using the Apexum procedure, seems to enhance healing kinetics with no adverse events.
